# TRANSLATION AND CULTURAL ADAPTATION OF THE MEDITERRANEAN DIET QUALITY INDEX IN CHILDREN AND ADOLESCENTS

**DOI:** 10.1590/1984-0462/2020/38/2018242

**Published:** 2020-01-13

**Authors:** Miriam Isabel Souza dos Santos Simon, Gabriele Carra Forte, Paulo Jose Cauduro Marostica

**Affiliations:** a Universidade Federal do Rio Grande do Sul, Porto Alegre, RS, Brazil.

**Keywords:** Adaptation, Mediterranean diet, Reproducibility of results, Adaptação, Dieta mediterrânea, Reprodutibilidade dos testes

## Abstract

**Objective:**

To translate and culturally adapt the Mediterranean Diet Quality Index in Children and Adolescent (KIDMED) for the Brazilian population.

**Methods:**

The processes of translation and cultural adaptation followed internationally standardized methodological norms. We used the intraclass correlation coefficient and the Bland-Altman dispersion analysis to assess the reproducibility and calculated the internal consistency with Cronbach’s alpha coefficient.

**Results:**

A total of 102 children and adolescents participated in the cross-cultural adaptation, of whom 58 (56.9%) were females, with a mean age of 9.8±4.9 years. The mean overall scores of adherence to the Mediterranean diet in the test and retest were similar (8.00 and 3.80 versus 8.01 and 3.84) for children and adolescents, respectively. The intraclass correlation coefficient for children and adolescents was 0.893 and 0.998, respectively. The internal consistency was 0.72. The Bland-Altman plot analysis showed good agreement between the final scores of the test and retest questionnaires, with no statistically significant difference.

**Conclusions:**

The KIDMED questionnaire was translated into Brazilian Portuguese and culturally adapted, presenting high reproducibility. This questionnaire can, therefore, be included and used in Brazilian studies that aim at evaluating the adherence to the Mediterranean diet among children and adolescents.

## RESUMO

**Objetivo:**

Traduzir e adaptar culturalmente a escala do *Mediterranean Diet Quality Index in Children and Adolescent* (KIDMED) para a população brasileira.

**Métodos:**

Os processos de tradução e adaptação cultural seguiram normas metodológicas padronizadas internacionalmente. A reprodutibilidade foi avaliada pelo coeficiente de correlação intraclasse e pela análise de dispersão de Bland-Altman, e a consistência interna foi calculada pelo coeficiente de Cronbach.

**Resultados:**

Participaram da etapa de adaptação transcultural 102 crianças e adolescentes, sendo 58 (56,9%) meninas com idade média de 9,8±4,9 anos. Os escores totais médio de aderência à dieta mediterrânea no teste e no reteste foram semelhantes (8,00 e 3,80 ***versus*** 8,01 e 3,84) para as crianças e os adolescentes, respectivamente. O coeficiente de correlação intraclasse para as crianças e os adolescentes foi de 0,893 e 0,998, respectivamente. A consistência interna obtida foi de 0,72. A análise do gráfico de Bland-Altman demonstrou boa concordância entre os escores finais dos questionários de teste e de reteste, sem diferença estatisticamente significativa.

**Conclusões:**

O questionário KIDMED foi traduzido e adaptado culturalmente para a língua portuguesa do Brasil, apresentando alta reprodutibilidade. Esse questionário pode ser, portanto, incluído e utilizado em estudos brasileiros que visem avaliar a aderência à dieta mediterrânea de crianças e adolescentes.

## INTRODUCTION

In recent decades, the dietary pattern of the Brazilian population has undergone rapid and significant changes, especially among children and adolescents. This change, characterized by the excessive consumption of processed and obesogenic foods, associated with the drastic reduction in energy expenditure, explains, in part, the epidemic proportion of chronic non-communicable diseases (NCDs) in adulthood.^1^

The Mediterranean dietary pattern has been considered one of the healthiest food models in the world, related to lower rates of chronic diseases and increased life expectancy. It has shown favorable effects on plasma lipoprotein concentrations, endothelium vasodilation, insulin resistance, metabolic syndrome, antioxidant capacity, and mortality from cardiovascular causes, possibly due to its anti-inflammatory effect.^2-4^

Although the Mediterranean Basin covers several different regions, all their diets share common characteristics, including the high intake of fruits, vegetables, and whole grains, the frequent consumption of oilseeds, the preference for white meat (fish and poultry) instead of red meat, and the low ingestion of saturated fat and cholesterol. Also, the consumption of foods rich in antioxidant and potentially anti-inflammatory components is encouraged.^5,6^

The Mediterranean Diet Quality Index in Children and Adolescents (KIDMED) is a questionnaire originally written in English and internationally accepted for the evaluation of adherence to the Mediterranean diet among children and adolescents. This questionnaire was inspired by a previous instrument developed for the adult^7^ and older adult^8^ populations.

In Brazil, we still do not have tools to evaluate adherence to the Mediterranean diet, possibly due to this diet being different from the usual dietary pattern of our population. Therefore, to use KIDMED in the Brazilian population, the questionnaire needed to go through a process of linguistic translation and cultural adaptation in order to maintain the psychometric properties of the original version, available in the English language. Thus, this study aimed to translate and culturally adapt the KIDMED scale for the Brazilian population.

## METHOD

KIDMED is a questionnaire developed to quantify adherence to the Mediterranean diet among children and adolescents. This instrument comprises 16 questions based on the assessment of eating habits, following the principles that support and weaken the patterns of this diet. This questionnaire can be self-administered or administered by a health professional through an interview. Each question has a “yes” or “no” answer, and the possibilities of response vary between -1 (negative connotation regarding the Mediterranean diet) and +1 (positive connotation regarding the Mediterranean diet), as in the original instrument (Table 1). The total index ranges from 0 to 12, and the final score is classified into three levels:

- >8, ideal Mediterranean diet.
- 4–7, improvements needed to adjust the intake to the patterns of the Mediterranean diet.
- ≤3, very poor dietary quality.^9^

**Table 1:** Original version of the Mediterranean Diet Quality Index in Children and Adolescents.

	*Scoring*	
1.	+1	Takes a fruit or fruit juice every day
2.	+1	Has a second fruit every day
3.	+1	Has fresh or cooked vegetables regularly once a day
4.	+1	Has fresh or cooked vegetables more than once a day
5.	+1	Consumes fish regularly (at least 2–3 times per week)
6.	-1	Goes more than once a week to a fast-food (hamburger) restaurants
7.	+1	Likes pulses and eats them more than once a week
8.	+1	Consumes pasta or rice almost every day (5 or more times per week)
9.	+1	Has cereals or grains (bread etc.) for breakfast
10.	+1	Consumes nuts regularly (at least 2–3 times per week)
11.	+1	Uses olive oil at home
12.	-1	Skips breakfast
13.	+1	Has a dairy product for breakfast (yoghurt, milk etc.)
14.	-1	Has commercially baked goods or pastries for breakfast
15.	+1	Takes two yoghurts and/or some cheese (40 g) daily
16.	-1	Takes sweets and candy several times every day
17.	-1	Takes sugar-sweetened beverage (SBB) (one cup or more) every day

The processes of translation and cultural adaptation of the questionnaire followed internationally standardized methodological norms.^10^ After obtaining formal authorization from the main author of KIDMED, Lluis Serra-Majem, by e-mail, we proceeded to the stage of translating and culturally adapting the instrument. The stages followed the procedures described below.

Two independent translators fluent in English, who knew the objectives of the present study, translated the original version of KIDMED into Portuguese. Two nutritionists responsible for the study compared and reconciled both versions, developing a final questionnaire in Portuguese (first draft). Subsequently, an independent translator (English teacher), whose native language is English and who is fluent in the target language (Portuguese), back-translated the first draft into English. This translator had no access to the original instrument and the purpose of the research. This back-translation and both previous translations of KIDMED were taken into consideration to reach a consensus and develop the second draft. An evaluation committee consisting of four health professionals (two nutritionists, a pediatric pulmonologist, and the main author of the instrument) assessed the second draft, in English and Portuguese, checking the semantic (between words), idiomatic (equivalent expressions not found), experimental (appropriate words for the cultural context), and conceptual (validity of the concept explored and the events experienced by lay individuals) equivalence, leading to the elaboration of the final version of the instrument, which was used in the adaptation process. The main author suggested the inclusion of an assessment item due to the high consumption of sugary beverages by part of the Brazilian population. Thus, the Brazilian version of KIDMED has 17 assessment items.

The cultural adaptation process was developed based on the methodology proposed by Beaton et al.^10^ The final translated version of the instrument was administered to adolescents and mothers and/or guardians of children under 10 years to evaluate the level of understanding and cognitive equivalence of this version of KIDMED (cognitive pre-test). We assessed the interviewees’ difficulty in understanding each item of the questionnaire. Cross-cultural adaptation was necessary for questions classified as hard to understand by more than 15% of the interviewees. Therefore, the difficulties raised during the interviews led to the adaptation of these items for the administration of the final test.

The measurement properties used in the final test were reproducibility (test-retest) and internal consistency. This stage involved 102 individuals – 51 mothers and/or guardians and 51 adolescents. Reproducibility was evaluated with minimum and maximum intervals of 7 and 10 days, respectively, aiming at comparing the results obtained by the same examiner at different times.

The sample size for the process of cross-cultural adaptation followed the suggestion of Beaton et al.^10^ for the pre-test stage. For internal consistency, the sample calculation was performed by multiplying the number of questions by six.^11^

Sample composition followed criteria similar to those used by Serra-Majem et al.^9^ when elaborating the original questionnaire. The sample included 66 healthy children aged 2–9 years, represented by their mothers and/or guardians, of whom 15 participated in the pre-test and 51 in the final stage of the questionnaire, in addition to 66 healthy adolescents (10–18 years), of whom 15 participated in the pre-test and 51 in the final stage of the questionnaire. We used a convenience sample from public and private schools covering the capital and inland of the state. We selected mothers from a pedagogical project (*Projeto Semear*) in Porto Alegre, Rio Grande do Sul, and adolescents from a public school (Escola Estadual Professor Ulysses Cabral) in Antonio Prado, Rio Grande do Sul. Participants were randomly chosen without distinction regarding ethnicity, marital status, or gender. The exclusion criterion was the inability to answer the questionnaires properly due to cognitive limitations.

We conducted a descriptive analysis of the sample identification variables. Quantitative data are expressed as mean±standard deviation or as median (interquartile range), depending on the distribution of the variables. We assessed the reproducibility by using the intraclass correlation coefficient (ICC) and the Bland-Altman plot analysis, which allowed us to observe the mean differences and the extreme limits of agreement, with a bias calculated by the paired Student’s *t*-test. Internal consistency was estimated by Cronbach’s alpha coefficient, with minimum acceptable value of 0.7. Data were processed and analyzed using the statistical software *PASW*, version 18.0 for Windows (IBM). We adopted a 5% significance level.

The Research Ethics Committee (REC) of the Hospital de Clínicas de Porto Alegre approved the study protocol (under no. 62014016.0.0000.5327). The guardians signed the informed consent form, and the adolescents, the agreement form.

## RESULTS

In the pre-test, conducted with 15 mothers and/or guardians of children aged 2–9 years and 15 adolescents aged 10–18 years, none of the interviewees refused to answer the questions or interrupted their participation. Semantic equivalence, which consists of grammar and vocabulary equivalence, was achieved with some changes; for instance, the word “pastries” was replaced for “baker’s confections” in question 14. Examples such as “beans, lentils, and peas” were included in parentheses after “pulses” in question 7. Similarly, we added “chestnuts, walnuts, peanuts, etc.” after “oilseeds” in question 10 as examples. Lastly, we included the words “soft drinks, juice boxes, processed juices, chocolate milk” after “sugar-sweetened beverages” in question 17. Thus, four questions were culturally adapted, concluding the final version of the instrument to be validated, as shown in Table 2.

**Table 2:** Version of the Mediterranean Diet Quality Index in Children and Adolescents translated into Portuguese.

	ESCORE	
1.	+1	Ingere uma fruta ou suco de fruta natural todos os dias
2.	+1	Ingere uma segunda fruta todos os dias
3.	+1	Ingere vegetais crus ou cozidos regularmente uma vez ao dia
4.	+1	Ingere vegetais crus ou cozidos mais de uma vez ao dia
5.	+1	Consome peixe regularmente (pelo menos 2–3 vezes por semana)
6.	-1	Vai mais que uma vez por semana em restaurantes ***fast-food*** (hambúrguer)
7.	+1	Ingere leguminosas (feijão, lentilha, ervilha) mais de uma vez por semana
8.	+1	Consome massa ou arroz quase todos os dias (cinco ou mais vezes por semana)
9.	+1	Ingere cereais ou grãos (pães etc.) no café da manhã
10.	+1	Consome oleaginosas (castanhas, nozes, amendoim etc.) regularmente (pelo menos 2–3 vezes por semana)
11.	+1	Usa azeite de oliva em casa
12.	-1	Não toma o café da manhã
13.	+1	Ingere um produto lácteo no café da manhã (iogurte, leite etc.)
14.	-1	Consome salgados assados ou doces de padaria no café da manhã
15.	+1	Ingere dois iogurtes e/ou duas fatias de queijo ao dia
16.	-1	Ingere doces e balas várias vezes ao dia
17	-1	Ingere um ou mais copos de bebidas açucaradas (refrigerantes, sucos de caixinha, sucos artificiais, achocolatado) por dia

A total of 102 children and adolescents participated in the cross-cultural adaptation, of whom 44 (43.1%) were males and 58 (56.9%), females, with a mean age of 9.8±4.95 years. The age of the children ranged from 2.03 to 9.17 years, and of the adolescents, from 10.89 to 18.51 years. Table 3 presents the characteristics of these children and adolescents. The mean age of the mothers and/or guardians was 36.61±5.01 years, ranging from 22 to 46 years. Regarding schooling, 36 (70.6%) mothers and/or guardians had complete higher education; 6 (11.8%), incomplete higher education; 7 (13.7%), complete high school; and only 2 (3.9%), incomplete high school.

**Table 3:** Characteristics of the 102 children and adolescents who participated in the final stage of the validation of the questionnaire.

Variables	Children (n=51)	Adolescents (n=51)
Gender, n (%)		
Male	14 (27.5)	30 (58.8)
Female	37 (72.5)	21 (41.2)
Mean age (years) ± standard deviation	5.29±2.03	14.33±1.96
Schooling (years), median [P25–P75]	0 [0–2]	8 [7–10]

n: sample size; P: percentile.

The mean overall scores of adherence to the Mediterranean diet in the test and retest were similar (8.0 and 3.8 versus 8.01 and 3.84) for children aged 2–9 years and adolescents, respectively.

The Brazilian version of KIDMED was considered easy and quick to administer, both to mothers and/or guardians and adolescents, with filling time ranging between two and seven minutes. The mean response time for mothers and/or guardians was 3±1 minutes, and for adolescents, 5±1 minutes.

**Figure 1:**
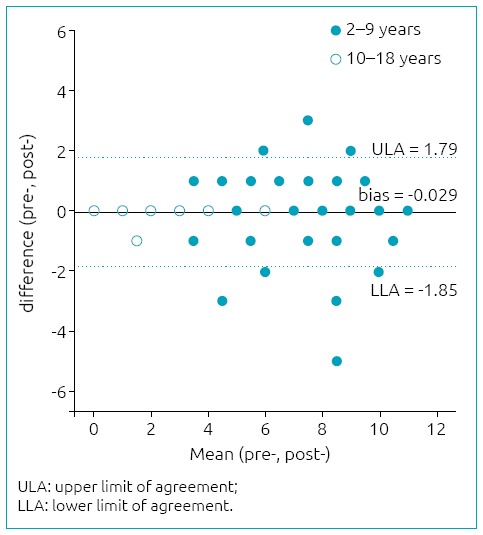
Agreement of overall score measures for the Mediterranean Diet Quality Index in Children and Adolescents in the test-retest according to the Bland-Altman analysis.

Table 4 shows the reproducibility analysis of the Brazilian version of KIDMED. The internal consistency obtained through Cronbach’s alpha coefficient was 0.72. The Bland-Altman plot analysis showed good agreement between the final scores of the questionnaires (Figure 1), with bias close to zero and no statistically significant difference (bias=0.029; p=0.749). The limits of agreement indicate that the difference between the two administrations of the questionnaires was lower than two points.

**Table 4:** Reproducibility (intraclass correlation coefficient) of the final score of the Mediterranean Diet Quality Index in Children and Adolescents administered to adolescents aged 10–18 years and mothers of children aged 2–9 years.

Age group	ICC	(95%CI)
2–9 years	0.893	(0.812–0.939)
10–18 years	0.998	(0.997–0.999)

ICC: intraclass correlation coefficient; 95%CI: 95% confidence interval.

## DISCUSSION

The translation of the original version of KIDMED into Portuguese and its cultural adaptation presented no difficulties. In the pre-test stage, the mothers and/or guardians and the adolescents understood most questions, and only four items from the original translation needed adjustments. In addition, the Brazilian version of KIDMED had good performance regarding filling time, proving to be a quick and practical tool.

According to some authors,^12,13^ the accuracy of a questionnaire assessed by Cronbach’s alpha coefficient must present values higher than 0.70 to ensure good internal consistency. Thus, the Brazilian version of KIDMED showed satisfactory results, attesting to its accuracy.

The ICC was higher among adolescents than among children, revealing excellent reproducibility in both groups (ICC above 0.75).

Concerning the adherence to the Mediterranean diet, the present study demonstrated that children aged 2–9 years had better dietary patterns compared to adolescents. On the other hand, the group aged 2–9 years showed greater variability in the dispersion analysis and the magnitude of differences between the questionnaires than adolescents. One explanation for these findings is the fact that the questionnaires for children were answered by their mothers or guardians, who may not know exactly what was consumed at school. Besides, the sample was recruited from a project in which most mothers had higher education, justifying, perhaps, the concern with a more diverse dietary pattern for their children. In contrast, the adolescents did not follow a healthy dietary pattern according to the Mediterranean diet but showed better regularity in their responses than children. Correa et al. analyzed 631 children and adolescents enrolled in public schools of Rio Grande do Sul and associated a healthy dietary pattern (high consumption of salads, cooked vegetables, fruits, beans, and milk/yogurt and low consumption of fried foods, hamburgers, cold cuts, crackers, snacks, cookies, hard candies, sweets, chocolates, and soft drinks) with children, and a restricted dietary pattern (low intake of all food groups and greater consumption of beans and soft drinks) with adolescents.^14^ Childhood and adolescence are fundamental to the development and establishment of eating habits. Adopting the Mediterranean dietary pattern in these life stages can contribute to reducing risk factors related to the onset of chronic NCDs in adulthood.

The present study has some limitations regarding the cultural adaptation of the items, considering the heterogeneous aspect of the Brazilian population; therefore, we recommend administering this questionnaire in different regions of Brazil. On the other hand, the benefits of this study should be taken into account, as it made available an instrument that assesses pediatric adherence to a diet that has been widely recommended.

We can conclude that the KIDMED questionnaire was translated into Portuguese and culturally adapted, presenting high reproducibility. Thus, it can be included and used in studies that aim at evaluating the adherence to the Mediterranean diet among Brazilian children and adolescents. Further studies to validate the translated version of KIDMED are fundamental to promote its wide use in different Brazilian contexts and populations.
